# A Comprehensive System for Generation and Evaluation of Induced Pluripotent Stem Cells Using piggyBac Transposition

**DOI:** 10.1371/journal.pone.0092973

**Published:** 2014-03-25

**Authors:** Tomoyuki Tsukiyama, Megumi Kato-Itoh, Hiromitsu Nakauchi, Yasuhide Ohinata

**Affiliations:** 1 PRESTO, Japan Science and Technology Agency (JST), Kawaguchi, Saitama, Japan; 2 Laboratory for Pluripotent Stem Cell Studies, RIKEN Center for Developmental Biology (CDB), Kobe, Hyogo, Japan; 3 Division of Stem Cell Therapy, Center for Stem Cell Biology and Regenerative Medicine, Institute of Medical Science, University of Tokyo, Minato-ku, Tokyo, Japan; 4 JST, ERATO, Nakauchi Stem Cell and Organ Regeneration Project, Chiyoda-ku, Tokyo, Japan; 5 Life Science Experimental Facility, Department of Biotechnology, Faculty of Life and Environmental Science, University of Yamanashi, Kofu, Yamanashi, Japan; Baylor College of Medicine, United States of America

## Abstract

The most stringent criterion for evaluating pluripotency is generation of chimeric animals with germline transmission ability. Because the quality of induced pluripotent stem cell (iPSC) lines is heterogeneous, an easy and accurate system to evaluate these abilities would be useful. In this study, we describe a simple but comprehensive system for generating and evaluating iPSCs by single transfection of multiple piggyBac (PB) plasmid vectors encoding Tet-inducible polycistronic reprogramming factors, a pluripotent-cell–specific reporter, a constitutively active reporter, and a sperm-specific reporter. Using this system, we reprogrammed 129 and NOD mouse embryonic fibroblasts into iPSCs, and then evaluated the molecular and functional properties of the resultant iPSCs by quantitative RT-PCR analysis and chimera formation assays. The iPSCs contributed extensively to chimeras, as indicated by the constitutively active TagRFP reporter, and also differentiated into sperm, as indicated by the late-spermatogenesis–specific Acr (acrosin)-EGFP reporter. Next, we established secondary MEFs from E13.5 chimeric embryos and efficiently generated secondary iPSCs by simple addition of doxycycline. Finally, we applied this system to establishment and evaluation of rat iPSCs and production of rat sperm in mouse–rat interspecific chimeras. By monitoring the fluorescence of Acr-EGFP reporter, we could easily detect seminiferous tubules containing rat iPSC–derived spermatids and sperm. And, we succeeded to obtain viable offspring by intracytoplasmic sperm injection (ICSI) using these haploid male germ cells. We propose that this system will enable robust strategies for induction and evaluation of iPSCs, not only in rodents but also in other mammals. Such strategies will be especially valuable in non-rodent species, in which verification of germline transmission by mating is inefficient and time-consuming.

## Introduction

To evaluate the characteristics and qualities of pluripotent stem cells, such as embryonic stem cells (ESCs) and induced pluripotent stem cells (iPSCs), several assays are used in combination. Among these assays, the most precise is verification of germline transmission by mating, because pluripotent stem cells are often utilized to generate genetically modified animal lines. However, this assay requires considerable effort and time: the process takes about 3 months in mouse, and longer in other animals. Furthermore, it is difficult to confirm germline transmission when the contribution ratio to the germline is low. To date, there has been no formal evidence that non-rodent pluripotent stem cells can contribute to the germline. Therefore, an easy and accurate system for assaying germline competency without mating would provide a valuable tool for evaluation of the characteristics and qualities of iPSC lines in non-rodent species.

iPSCs are generated from somatic cells by transient transduction of four defined transcription factors, termed the reprogramming factors: Oct3/4, Sox2, Klf4, and c-Myc [1]. The quality of the resultant iPSC lines is heterogeneous, and only some cell lines are germline-competent [2], [3]. Therefore, it would be valuable to develop a method for evaluating the germline competency of iPSCs more easily and accurately than is currently possible using conventional methods. Because the piggyBac (PB) system enables simultaneous and highly efficient insertion of multiple exogenous genes into a genome, use of this system allows reprogramming factors and additional reporter systems to be introduced into cells at the same time [4]–[7]. PB transposition takes place in DNA regions flanked by terminal repeat sequences; therefore, inserted DNAs tend to contain full sequences. By contrast, portions of exogenous DNA sequences are often lost in transgenic animals produced by conventional methods that involve plasmid injection into embryos. Although viral vector systems, such as lentiviral systems, also can transfer full sequences flanked by terminal repeats, such methods require special equipment and techniques. Because the PB system is a non-viral system based on a DNA transposon mechanism, no special methods are necessary. Instead, the PB system requires only a simple procedure consisting of a single transfection with multiple plasmids. Previously, we described a simple and efficient method for generating iPSCs by piggyBac transposition [8]. In this study, we modified our previous method to design a comprehensive and non-invasive system for generation and evaluation of iPSCs using simultaneous piggyBac transpositions of reprogramming factors and multiple fluorescent reporters. The multiple-reporter system has three components: an EOS (early transposon promoter and *Oct3/4* and *Sox2* enhancers)-EGFP reporter [8], [9] to detect pluripotent stem cells, a constitutively active CAG-TagRFP reporter [8], [10] to detect iPSC-derived cells in chimeric animals, and an acrosin reporter [11] to detect iPSC-derived spermatid and sperm in chimeric animals. Acrosin is expressed specifically in late spermatogenesis, and accumulates in acrosomes in spermatids and sperm [12]. The acrosin reporter construct encodes an acrosin N-terminal signal peptide fused to EGFP, controlled by the acrosin promoter (Acr-EGFP) [11]. This reporter enables detection of intact, living, mature male germ cells under a fluorescence microscope and recovery of these cells for applications such as intracytoplasmic sperm injection (ICSI).

Several groups have developed an efficient reprogramming system, called the secondary reprogramming system, consisting of somatic cells derived from chimeric mice using tetracycline (Tet)-inducible iPSCs [13]–[15]. In this system, the donor cells for iPSC induction already contain Tet-inducible genes encoding the reprogramming factors. Therefore, iPSCs can be generated by simple addition of doxycycline (Dox, a member of the tetracycline antibiotics group) to the culture media. Our system also contains Tet-inducible reprogramming factors, enabling establishment of a secondary reprogramming system.

Finally, we generated rat iPSCs from native rat embryonic fibroblasts and evaluated their ability to contribute to mouse development. Recently, two groups succeeded in generating rat–mouse chimeras and demonstrating that rat iPSCs were able to rescue a genetic deficiency in the host mouse blastocysts, resulting in rat organs that appeared normal by functional complementation criteria [16], [17]. In particular, Isotani et al. confirmed the contribution of rat ESCs to spermatogenesis in testes of mouse–rat chimeras, as revealed by their characteristic morphology [17]. To date, however, no report has demonstrated germline contribution of iPSCs in mouse–rat interspecific chimeras. This is the first report to form functional sperm from rat iPSCs in mouse–rat interspecific chimeras.

In this paper, we demonstrate that our system can generate germline-competent iPSCs in mouse and rat, and that we can evaluate the formation of iPSC-derived male germ cells in the testes of chimeric animals using the Acr-EGFP reporter.

## Results

### Generation and evaluation of mouse iPSCs

**Figure 1:**
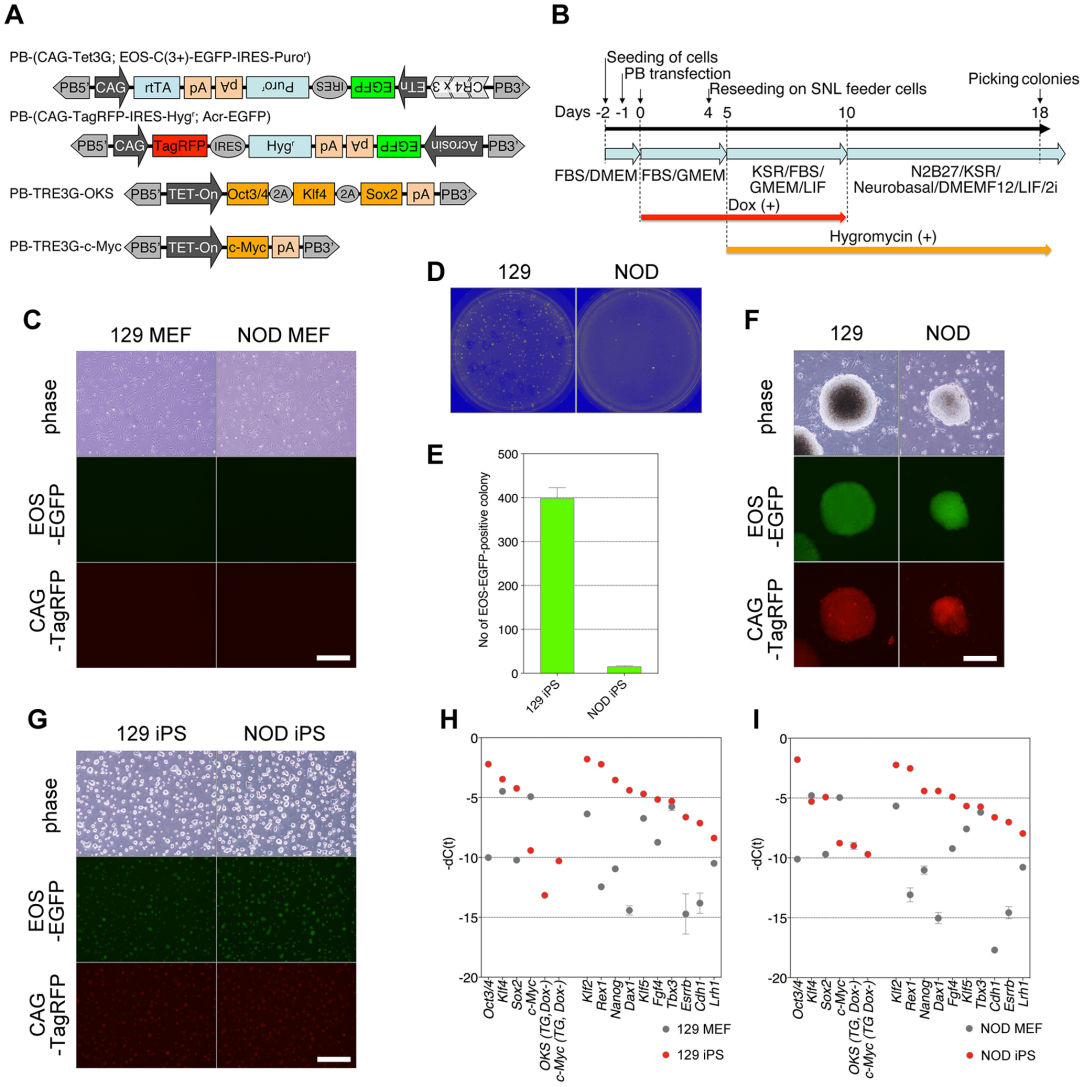
Reprogramming of mouse somatic cells and establishment of iPSCs. (A) Construction of PB vectors used in this study. (B) Timeline for the establishment of iPSC lines. (C) Morphology of donor MEFs. Scale bar, 50 μm. (D and E) Numbers of primary iPSC colonies. (F) Morphology of primary iPSC colonies. Scale bar, 50 μm. (G) Morphology of iPSC cell lines. Scale bar, 50 μm. (H and I) Endogenous expression levels of the four reprogramming factors, transgenes, and ESC-specific genes in MEFs and iPSCs.

In order to effectively generate and evaluate iPSCs, we first constructed a series of new vectors (Fig. 1A). The pCAG-hyPBase vector expresses a hyperactive piggyBac transposase under the control of the CAG promoter [18]. The PB-(CAG-Tet3G; EOS-C(3+)-EGFP-IRES-puro^r^) vector expresses a reverse trans-activator (rtTA) under the control of the CAG promoter, and also contains an EOS-EGFP reporter and a puromycin-resistance gene as markers for pluripotency. The PB-TRE3G-OKS and PB-TRE3G-c-Myc vectors express the reprogramming factors (Oct3/4, Klf4, Sox2, and c-Myc) under the control of a Tet-inducible promoter. The PB-(CAG-TagRFP-IRES-hyg; Acr-EGFP) vector expresses TagRFP and a hygromycin resistance gene under the control of a constitutively active CAG promoter in most types of cells, as well as a spermatid- and spermatozoa-specific EGFP reporter under the control of the acrosin gene promoter.

NOD and 129 mouse embryonic fibroblasts (MEFs) were transfected with all of these vectors simultaneously and cultured in FBS/GMEM containing Dox (Fig. 1B, C). Four days after Dox addition, 5×10^5^ cells were reseeded on an SNL feeder layer, and the medium was replaced with the LIF medium the next day. Ten days after Dox addition, Dox was withdrawn, and the medium was replaced with the LIF/2i medium. After an additional 8 days of culture without Dox, a large number of EOS-GFP–positive ES-like colonies appeared (Fig. 1D, E, F). EOS-GFP– and CAG-TagRFP–positive colonies were picked and reseeded on feeder cells. After one passage, the cells were cultured without feeders. We efficiently established iPSC lines from MEFs from both the 129 and NOD strains. These iPSC lines co-expressed CAG-TagRFP and EOS-EGFP (Fig. 1G). Quantitative real-time RT-PCR analysis showed that these iPSCs expressed undifferentiated ESC–specific genes, including *Oct3/4, Klf4*, *Sox2*, *Klf2*, *Rex1*, *Nanog*, *Dax1*, *Klf5*, *Fgf4*, *Tbx3*, *Esrrb*, *Cdh1*, and *Lrh1* (Fig. 1H, I). This analysis showed that exogenous expression of the reprogramming factors in these iPSCs was efficiently downregulated to background levels following Dox withdrawal (Fig. 1H, I).

**Figure 2:**
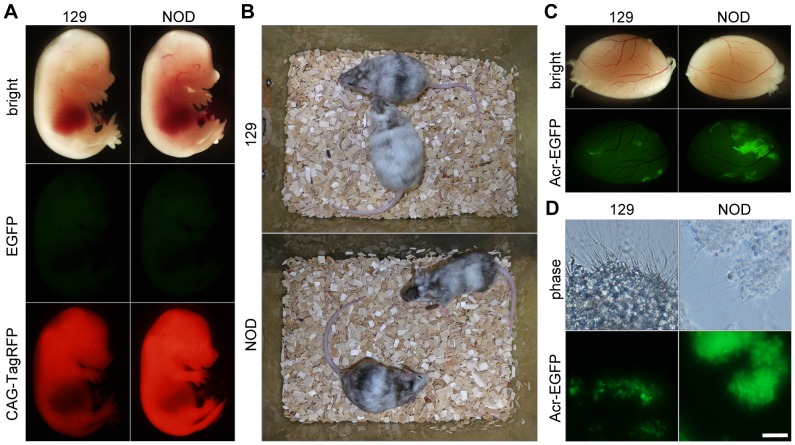
Evaluation of established mouse iPSC lines. (A) Contribution of iPSCs to mouse embryonic development. Embryos were analyzed by fluorescence microscopy at E13.5. (B) Coat color of adult chimeras at 7 weeks old. (C) Contribution of iPSCs to mouse testes. (D) EGFP-positive sperm and spermatids. Scale bar, 5 μm.

To evaluate the ability of these iPSC lines to contribute to chimeric animals, we injected 129 and NOD iPSCs into CD-1 and C57BL/6 mouse blastocysts, respectively, and visualized the contribution of donor cells to the resultant chimeric embryos by monitoring CAG-TagRFP fluorescence at embryonic day (E) 13.5 (Fig. 2A). Embryos (20 from 129 iPSCs, and 48 from NOD iPSCs) were transferred into pseudopregnant mice. From those implantations, 19 live 129 embryos and 10 live NOD embryos were obtained from two pseudopregnant mice at E13.5. Eleven of the 129 embryos and seven of the NOD embryos were chimeras, as determined by their RFP fluorescence and eye color. The coat color of adult chimeras also demonstrated the ability of these iPSCs to contribute to chimeric animals (Fig. 2B). In order to assess the germline competency of these iPSC lines, we examined the testes of chimeras at 7 weeks of age (Fig. 2C). The acrosomes of spermatids and spermatozoa derived from donor iPSCs could be specifically distinguished by monitoring fluorescence from the Acr-EGFP reporter (Fig. 2D).

**Figure 3:**
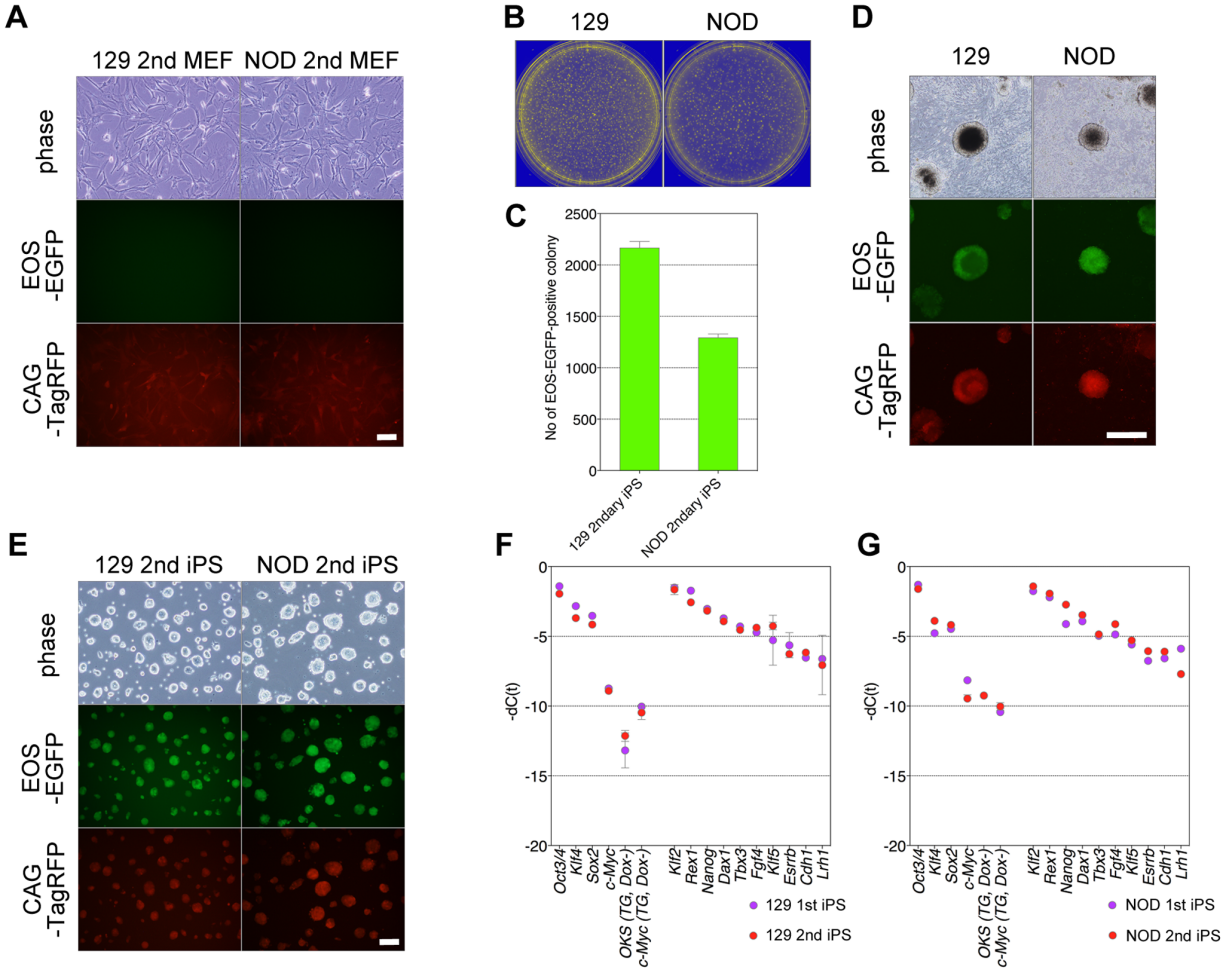
Establishment of secondary MEFs and secondary iPSCs. (A) Morphology of secondary MEFs. Scale bar, 10 μm. (B and C) Numbers of secondary iPSC colonies. (D) Morphology of secondary iPSC colonies. Scale bar, 50 μm. (E) Morphology of secondary iPSC cell lines. Scale bar, 10 μm. (F and G) Comparison of endogenous expressions of the four reprogramming factors, transgenes, and ESC-specific genes between primary and secondary iPSCs.

We then collected these chimeric embryos and trypsinized them. After several passages under hygromycin selection to isolate donor-derived cells, we obtained secondary MEFs containing the previously transfected vectors (Fig. 3A) [13]–[15]. Using these secondary MEFs, we could efficiently generate secondary iPSCs by Dox addition alone, without additional transfection (Fig. 3B, C, D). The secondary iPSC lines expressed both TagRFP and EOS-EGFP (Fig. 3E). Quantitative real-time RT-PCR analysis showed that the expression levels of the exogenous reprogramming factors were efficiently downregulated in these secondary iPSCs in the absence of Dox compared to the expression levels of endogenous transcription factors, and these cells expressed many undifferentiated ESC–specific genes, including *Oct3/4, Klf4*, *Sox2*, *Klf2*, *Rex1*, *Nanog*, *Dax1*, *Tbx3*, *Fgf4*, *Klf5*, *Esrrb*, *Cdh1*, and *Lrh1* (Fig. 3F, G).

### Generation and evaluation of rat iPSCs

**Figure 4:**
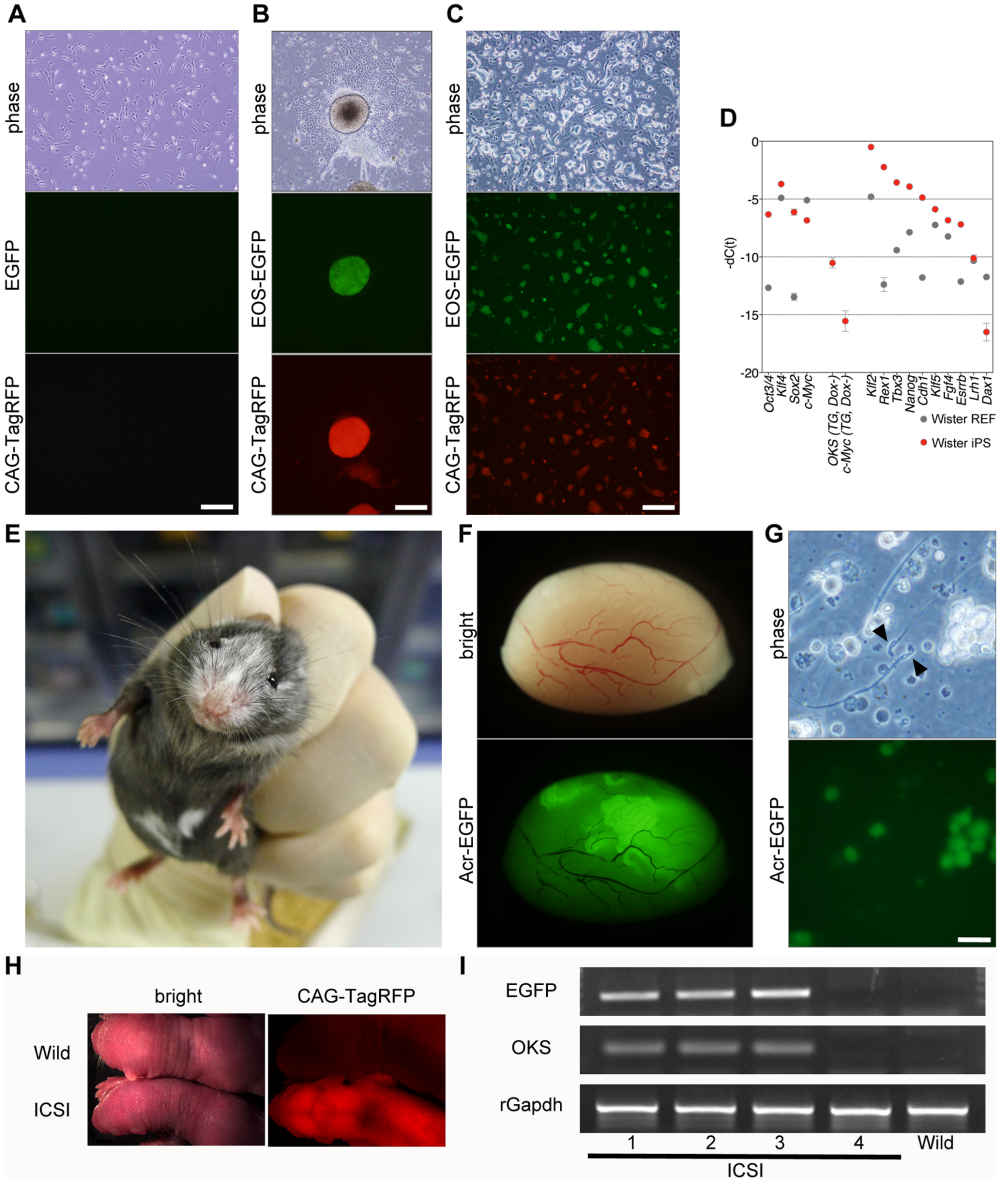
Establishment of rat iPSCs and production of rat iPSC–derived sperm in mouse–rat interspecific chimeras. (A) Morphology of REFs. Scale bar, 50 μm. (B) Morphology of rat iPSC primary colonies. Scale bar, 50 μm. (C) Morphology of rat iPSCs. Scale bar, 50 μm. (D) Endogenous expression levels of the four reprogramming factors, transgenes, and ESC-specific genes in REFs and rat iPSCs. (E) Coat color of adult rat–mouse interspecific chimeras at 8 weeks of age. (F) Contribution of rat iPSCs to mouse testes. (G) Rat sperm in EGFP-positive seminiferous tubules of mouse–rat interspecific chimeras. Scale bar, 5 μm. (H) A rat offspring produced by ICSI and a wild-type rat offspring. (I) Genomic PCR of rat offspring.

Using the same system, we established rat iPSCs from rat embryonic fibroblasts (REFs) (Fig. 4A, B). These rat iPSCs expressed TagRFP and EOS-EGFP (Fig. 4C). In these rat iPSCs, the expression levels of the exogenous reprogramming factors were efficiently downregulated in the absence of Dox compared to the expression revels of the endogenous transcription factors, and these cells expressed many undifferentiated ESC–specific genes, including *Oct3/4, Klf4*, *Sox2*, *Klf2*, *Rex1*, *Tbx3*, *Nanog*, *Cdh1*, *Klf5*, *Fgf4*, and *Esrrb* (Fig. 4D). Unexpectedly, *Dax1* and *Lrh1* were not expressed in these cells (Fig. 4D).

To examine the ability of these rat iPSCs to contribute to chimeric animals, we generated mouse–rat chimeric animals by injection of rat iPSCs into mouse blastocysts. Forty-four embryos were transferred into pseudopregnant mice, resulting in four chimeras, as determined by their coat color (Fig. 4E).

To evaluate the ability of the rat iPSCs to contribute to the germline, we examined the testes of mouse–rat chimeras at 8 weeks of age. Although the chimeric ratio was not high, as determined by coat color (approximately 10–15%), EGFP-positive seminiferous tubules were easily detected (Fig. 4F). Of six testes we examined, two had EGFP-positive seminiferous tubules. In these seminiferous tubules, only rat-iPSC–derived spermatogenic cells expressed EGFP (Fig. 4G). We were able to distinguish rat and mouse sperm based on morphological differences of the sperm heads (Fig. 4G). Unexpectedly, however, mouse acrosin-EGFP fusion protein did not accumulate in the acrosomes of the rat haploid germ cells. Finally, to examine the ability of these rat sperm to produce pups, we performed ICSI and injected the sperm heads into 41 oocytes. The experiments demonstrated that four viable rat offspring with red fluorescence could be obtained from 29 transferred eggs (Fig. 4H). Genomic PCR analysis also showed the presence of vector-derived EGFP gene and OKS gene in three of the four offspring, as expected from the hemizygosity of the transgenes (Fig. 4I).

## Discussion

In this paper, we describe a simple but comprehensive system for generating and evaluating iPSCs by a single transfection of multiple PB plasmid vectors. This system enables us to evaluate iPSCs for their pluripotency, establish secondary iPSC-generation systems, and verify the ability of iPSCs to contribute to chimeras and to transmit their genes through the germline, accurately and without needing to check the coat color of F1 offspring. The multiple reporters used in this system allow the abilities of iPSCs to be evaluated in several ways. First, we can evaluate iPSCs by checking their morphology after Dox withdrawal. After Dox withdrawal, expression of transgenes is eliminated; therefore, only cells proliferating without the support of transgenes can form colonies. Second, we can easily identify iPSCs by monitoring fluorescence of the EOS-GFP-reporter [9]. In our system, the EOS reporter and rtTA are on the same vector; therefore, all cells in colonies that emerge during the reprogramming process are expected to harbor at least one EOS reporter. Third, we can evaluate the differentiation capacity of iPSCs by monitoring the fluorescence of the TagRFP reporter in chimeras. This enables us to evaluate these cells' ability to contribute to chimeras at early time points of gestation. Our results show that we could easily identify chimeric embryos based on their bright red fluorescence during embryogenesis. Although the contribution ratio of NOD iPSCs to chimeras was high, that of 129 iPSCs was relatively low, possibly due to differences in the mouse strains used as blastocyst hosts. Finally, we can evaluate the ability of iPSCs to differentiate into germ cells by monitoring the fluorescence of the acrosin-EGFP-reporter [11]. iPSC-derived mouse and rat sperm could be easily distinguished from host cells by their EGFP fluorescence, even when the contribution ratio was low. The expression of EGFP was limited to spermatogenetic cells and sperm acrosomes, indicating that the specificity of this PB reporter system was comparable to that of conventional transgenic-reporter mouse strains [11], [19].

In general, production of transgenic animals via plasmid injection into male pronuclei of fertilized eggs often yields animals that lack portions of vector sequences. Here, we applied the PB system to introduce full sequences of reporter transgene into somatic cells, and reproducibly succeeded in obtaining reporter-transgenic iPSCs that recapitulated endogenous gene expression in chimeric animals. This result indicates that our system can efficiently and accurately produce reporter-transgenic cells and animals.

In many studies of iPSC reprogramming, transgenic cell lines that already harbored pluripotency-specific reporters such as Oct3/4-GFP, Nanog-GFP, or Sox2-GFP have been used as donor cells in order to allow detection of successfully reprogrammed cells [2], [14], [20], [21]. By contrast, in this study we were able to easily establish iPSC lines from fibroblasts not previously subjected to genetic manipulation, not only in mice but also in rats. Because it is time-consuming and costly to produce transgenic animals and prepare transgenic cell lines, our strategy should be especially useful in large mammals.

In this study, we were able to establish secondary MEFs and establish secondary iPSC lines from these cells. In our system, secondary donor cell–derived embryonic fibroblasts can be collected easily using hygromycin selection, because a hygromycin-resistance gene is introduced into cells along with TagRFP. Therefore, this system enables establishment of secondary embryonic fibroblasts, even if the contribution ratio is quite low. To date, secondary reprogramming systems have been reported only in mice and humans [13]–[15], [22], [23]. We propose that our system will be useful for establishing secondary system in a range of mammalian species.

Finally, we were able to generate rat iPSCs by using the same system. These rat iPSCs contributed to the mouse–rat interspecific chimeras, including their germline. We were able to detect and isolate donor-derived sperm easily, although only spermatogenic cells expressed EGFP, whereas sperm did not exhibit EGFP fluorescence. This result indicates that localization of EGFP to acrosomes did not occur, even though EGFP was expressed accurately under the control of the acrosin promoter. This problem might be overcome by using the signal peptide and N-terminal peptide of rat proacrosin. Recently, Hanna's group reported derivation of human ground-state naïve pluripotent stem cells [24]; their approach may enable establishment of naïve pluripotent stem cells in various mammalian species. Because germline transmission is inefficient, time-consuming, and costly in non-rodent animals, we propose that this system will be useful for applications in larger mammals. Furthermore, in the case of interspecific chimeras, mating is difficult, so this strategy may be useful for germ-cell production from interspecific chimeric animals. Previously, we reported *in vitro* reconstitution of primordial germ cell (PGC) fate specification process from pluripotent epiblast [25]. Although *in vitro* production of sperm from PGCs has not been reported, it has been possible to produce sperm from spermatogonia in *in vitro* cultures of neonatal mouse testes [26]. Therefore, iPSCs harboring the Acr-EGFP reporter will be useful in studies of *in vitro* germ-cell development, and should allow non-invasive evaluation of *in vitro* haploid male germ-cell formation from somatic cells.

## Materials and Methods

### Vector construction

To generate PB-TRE3G-OKS and PB-TRE3G-c-Myc, pTRE3G-IRES (Clontech) was amplified by PCR and cloned into PB-hCMV*1-cHApA [27], [28] to yield PB-TRE3G-cHApA, and then inserts from PB-TET-OKS [8], [29] and PB-TET-c-Myc were introduced into PB-TRE3G-cHApA. To generate PB-(CAG-Tet3G; EOS-C(3+)-EGFP-IRES-puro), inserts from pCMV-Tet3G (Clontech) and PB-EOS-C(3+)-EiP [8], [9] were cloned into PB-CAG-rtTA_Adv [8], [29]. To generate PB-(CAG-TagRFP-IRES-hyg; Acr-EGFP), inserts from pAcr3-EGFP [11] was introduced into empty PB vector, and the resultant vector (PB-Acr-EGFP) was amplified by PCR and introduced into PB-CAG-cHA-IRES-hyg to yield PB-(CAG-cHA-IRES-hyg; Acr-EGFP); next, an insert from pTagRFP-N1 (Evrogen) was introduced into PB-(CAG-cHA-IRES-hyg; Acr-EGFP). To generate pCAG-hyPBase, pCMV-hyPBase [18] was cloned into blunt-ended pCAGGS [10]. Primer sequences are shown in Table S1.

### Cell culture

To obtain MEFs and REFs, we purchased 129/Sv mice and wistar rats from CLEA Japan, Inc. and Japan SLC, Inc., respectively. Embryos were collected at E13.5 and E15.5, respectively. After removal of heads and visceral tissues, the remaining bodies were washed in fresh PBS and trypsinized, and the isolated cells were maintained in Dulbecco's modified eagle medium (DMEM, Invitrogen) containing 10% fetal bovine serum (FBS, JRH Biosciences), penicillin, streptomycin (Invitrogen), and primocin (Invivogen).

To establish mouse or rat iPSC lines, 1×10^5^ MEFs or REFs were simultaneously transfected with 1 μg PB-TRE3G-OKS, 1 μg PB-TRE3G-c-Myc, 1 μg PB-(CAG-Tet3G; EOS-C(3+)-EGFP-IRES-puro), 1 μg PB-(CAG-TagRFP-IRES-hyg; Acr-EGFP), and 1 μg pCAG-hyPBase. Transfected cells were cultured in DMEM supplemented with 10% FBS. One day after transfection, the medium was replaced with Glasgow minimum essential medium (GMEM, Sigma) supplemented with 10% FBS, 1 mM sodium pyruvate (Invitrogen), 1×MEM non-essential amino acids (NEAA, Invitrogen), 0.1 mM 2-mercaptoethanol (2-ME, Millipore), 2 mM L-glutamine, penicillin, and streptomycin (FBS/GMEM) containing 1.5 μg/ml doxycycline (Dox, Sigma). Four days after Dox addition, 5×10^5^ cells were reseeded on an SNL feeder layer, and the medium was replaced the next day with GMEM supplemented with 15% Knockout Serum Replacement (KSR, Invitrogen), 0.3% FBS, 1 mM sodium pyruvate, 1×NEAA, 0.1 mM 2-ME, 2 mM L-glutamine, penicillin, streptomycin, and 1000 U/ml leukemia inhibitory factor (LIF, Millipore) (LIF medium) containing 200 μg/ml hygromycin (Sigma). In the case of secondary iPSC induction, 1×10^5^ transfected cells were reseeded on an SNL feeder layer, but hygromycin was not added to the medium. Ten days after Dox addition, Dox was withdrawn and the medium was replaced with 50% Neurobasal medium (Invitrogen) and 50% DMEM/F12 (Invitrogen) supplemented with 0.5×N2 (Invitrogen), 0.5×B27 (Invitrogen), 1% KSR, 0.05% BSA (Sigma), 0.15 mM 1-thioglycerol (Sigma), 2 mM L-glutamine, penicillin, streptomycin, 1000 U/ml LIF, 1 μM PD0325901, and 3 μM CHIR99021 (2i [30], Stemgent) (LIF/2i medium) containing 200 μg/ml hygromycin. After an additional 8 days of culture without Dox, CAG-TagRFP– and EOS-EGFP–double-positive primary colonies were picked, chemically dissociated, and transferred onto fresh SNL feeder layers in 48-well plates. For mouse iPSC generation, cells were cultured on human plasma fibronectin (Millipore) following passage. For rat iPSC generation, cells were maintained on SNL feeder layers on human plasma fibronectin or Matrigel (BD).

### Quantitative real-time RT-PCR

Total RNA was extracted from cells using RNeasy Mini kits (QIAGEN). For reverse transcription, ReverTra Ace (Toyobo) and oligo (dT)_20_ primer were used according to the manufacturer's instructions. For real-time PCR, Power SYBR Green PCR Master Mix (Applied Biosystems) was used. Transcription levels were determined in triplicate reactions and normalized to *Gapdh*. Primer sequences are shown in Table S1.

### Chimera production

To generate chimeric animals, mouse or rat iPSCs were injected into mouse embryos at the blastocyst stage. iPSCs from strain 129 were injected into CD-1 blastocysts. NOD and rat iPSCs were injected into C57BL/6 blastocysts. Blastocysts were transferred into the uterine horns of five pseudopregnant mice. Chimerism of live embryos and pups was judged by RFP fluorescence, eye color, and coat color. The contribution of Acr-EGFP-positive cells into the lumina of seminiferous tubules was analyzed on an inverted fluorescence microscope (Olympus).

### ICSI

Rat ICSI was performed according to the method described previously [31]. Briefly, oocytes were harvested from superovulated 4-week-old female rats (Slc∶SD). The sperm heads were injected into oocytes in Hepes-mR1ECM medium using micromanipulators (Narishige) and a piezo impact-driving unit (Prime Tech). The ICSI oocytes were cultured in 100-μl microdrops of mR1ECM/BSA medium and were transferred into the oviducts of 14-week-old pseudopregnant recipient rat (Crlj∶WI). The presence of TagRFP gene in the offspring was examined by fluorescence and the presence of EGFP gene and OKS gene were examined by genomic PCR. Primer sequences are shown in Table S1.

### Animal Ethics Statement

All animal experiments conformed to our Guidelines for the Care and Use of Laboratory Animals and were approved by the Institutional Committee for Laboratory Animal Experimentation (RIKEN Kobe Institute).

## Supporting Information

Table S1 **Primers for qRT-PCR.**
(XLSX)
