# Correction: A Comprehensive System for Generation and Evaluation of Induced Pluripotent Stem Cells Using piggyBac Transposition

**DOI:** 10.1371/journal.pone.0101922

**Published:** 2014-07-01

**Authors:** The PLOS ONE Staff

The affiliation for the second author is incorrect. Megumi Kato-Itoh is affiliated with #3 and #4, but not with #1. Her correct affiliation is:

Megumi Kato-Itoh^3,4^

3 JST, ERATO, Nakauchi Stem Cell and Organ Regeneration Project, Chiyoda-ku, Tokyo, Japan

4 Life Science Experimental Facility, Department of Biotechnology, Faculty of Life and Environmental Science, University of Yamanashi, Kofu, Yamanashi, Japan

There is a spelling error in the Acknowledgements. The pCMV-hyPBase vector was provided by Dr. Kosuke Yusa. The correct Acknowledgements statement is:

We are grateful to Dr. Hitoshi Niwa (RIKEN Center for Developmental Biology) for invaluable comments and advice on this work. We thank the following people for kindly providing materials for this study: Dr. Hitoshi Niwa for the empty PB vector and PB-CAG-cHA-IRES-hyg, Dr. Allan Bradley (Sanger Institute) for SNL feeder cells and Dr. Kosuke Yusa (Sanger Institute) for the pCMV-hyPBase vector.
